# Transcriptome dysregulation and epigenome signature responses in hepatocytes upon hepatitis B virus infection and therapy

**DOI:** 10.1186/1756-8935-6-S1-P67

**Published:** 2013-03-18

**Authors:** Andreas Klein, Lisa Wiluhn, Theodor Winkler, Timo Deba, Valerie Orth, Kai Hensel, Andreas Jenke, Stefan Wirth, Jan Postberg

**Affiliations:** 1 Zentrum für Kinder- und Jugendmedizin, HELIOS Klinikum Wuppertal, Universität Witten/Herdecke, Wuppertal, Germany

## Background

Chronic infection with hepatitis B virus (HBV) induces transregulation of the host cell gene expression and eventually malignant transformation. HBV X protein acts as a transactivator of cellular promoters leading to upregulation of DNA methyltransferases and alterations of DNA methylation patterns.

## Aim of the study

To contribute to our understanding of DNA and chromatin-modifying mechanisms modulated by HBV, which are responsible for the transregulation of host cells for virus particle production and malignant transformation of hepatocytes. We further study the effect of lamivudine treatment or an antiviral vector-based RNAi strategy targeting HBx on the establishment and maintenance of DNA methylation patterns. Our research not only aims to uncover epigenomic plasticity modulated by HBV. We also intend to contribute to the open problem, whether therapies lead to the restoration of regular epigenomic signatures, or whether hepatocytes persist in a deregulated epigenetic ‘memory’ state of HBV infection thus still carrying the risk for malignant transformation.

## Methods

Therefore we analyze expression profiles by qRT PCR arrays. DNA methylation as well as chromatin signatures of genes up- or downregulated upon HBV infection are studied by MeDIP or ChIP respectively, in murine cell lines infected with HBV.

## Results

We identified several transregulated genes and monitored DNA methylation and chromatin signatures at selected loci within promoters. Moreover we observed that therapeutic strategies lead to changes in the epigenomic signature of hepatocytes.

## Conclusions

Our data show that our experimental therapeutic interventions efficiently suppress HBV proliferation, whereas an increased risk of malignant transformation cannot be excluded.

